# The infant with bilateral skull fractures: diagnostic considerations in consultation with a child abuse pediatrician

**DOI:** 10.5249/jivr.v11i1.938

**Published:** 2019-01

**Authors:** Mandy A. O’Hara

**Affiliations:** ^*a*^Department of Pediatrics, New York Presbyterian Hospital, Columbia University Medical Center, New York, USA.

**Keywords:** Skull Fracture, Bilateral, Biparietal, Accident, Abuse

## Abstract

Bilateral skull fractures in infancy may result from accidental or abusive injury. Consultation with a child abuse pediatrician may assist with determining the likelihood of accident or abuse. Diagnostic considerations for the infant with bilateral skull fractures are reviewed, including single impact, double impact, and compression mechanisms of injury, as well as the possibility of accessory sutures as skull fracture mimics. Illustrative cases exemplify the evaluative process, including obtaining a detailed history, assessing for the presence or absence of additional physical or radiographic signs of injury, screening for psychosoical risk, and obtaining three-dimensional reconstruction of CT bone images. An understanding of plausible mechanisms of injury that can result in bilateral skull fractures in infancy can assist with making an accurate determination of likelihood of accident or abuse.

## Introduction

Skull fractures in infancy are common in both accidental trauma and inflicted injury.^1^ Simple linear fractures of the parietal bone are the most common type of skull fracture in both abusive injury and accidents. ^2,3^ However, certain fracture characteristics, including bilateral or multiple skull fractures, fractures crossing sutures, or depressed or diastatic in nature, are more likely associated with abuse. ^4,5,6,7^ The finding of bilateral skull fractures in an infant often prompts consultation with a child abuse pediatrician to assist with understanding the likelihood of abuse. When the mechanism of injury provided on history does not seem to be a plausible explanation of the injurious findings, concern for inflicted injury may lead to child pro-tection agency involvement and an investigation. Although often suspicious for abuse, some studies support that complex skull fractures, including bilateral fractures, can occur accidentally,^8,9^ even from a single impact event.^10,11^ It is thus crucial to understand all plausible mechanisms of injury as well as possible anatomic mimics, to accurately determine the mechanism of injury in an infant diagnosed with bilateral skull fractures.

This article reviews the diagnostic considerations when evaluating an infant with the finding of bilateral skull fractures. Mechanisms of injury will be discussed, with case examples highlighting the importance of a detailed history and physical exam, the presence or absence of any additional findings or risk factors con-cerning for abuse, as well as a thorough review of radiographic imaging. Consultation with a child abuse pediatrician may assist in determining if bilateral skull fractures in an infant are consistent with the injury as re-ported on history, or suspicious for abuse. In addition, anatomic variants that mimic fracture will be discussed and should always be considered.

**Mechanisms of injury**

**Double impact trauma**

Bilateral skull fractures can occur from two direct impact sites, such as two separate blows to the head in inflicted injury. Abusive head trauma, which usually involves violent shaking of an infant, can occur with or without impact of the head against a hard surface.^12^ If impact occurs on both sides of the head, bilateral abusive fractures can occur. However, studies show that the most cases of abusive head trauma involve traumatic brain injury without skull fracture alone, and the presence of skull fracture increases the likelihood of an accidental fall.^13^ If abusive head trauma is a diagnostic consideration, a complete evaluation, including intracranial imaging, dilated retinal exam, and complete skeletal survey should always be done, in particular for the young infant. If additional findings associated with abuse or inflicted head injuries are found, such as unexplained bruising on exam, rib fracture or other fracture, subdural hemorrhages, or retinal hemorrhages, a diagnosis of abuse is more likely. However, in the absence of any additional findings, accidental injury or anatomic variants must also be considered.

The mechanism and acuity of injury may be supported by soft tissue swelling overlying a fracture site. In order to best understand injury in an infant with bilateral skull fractures, a thorough evaluation involves closely assessing for external scalp injury and soft tissue swelling on exam and radiographic imaging, as external scalp lesions and soft tissue swelling may clinically indicate an impact site. Soft tissue swelling, from subcutaneous hemorrhage or edema, subgaleal hemorrhage, or a subperiosteal cephalohematoma, can appear immediately or within hours to days, with gradual resolution typically within 7-10 days.^9,19^ Although scalp swelling indicates acuity of injury within this time frame, in the absence of soft tissue swelling, skull fractures cannot be reliably dated.^14,19^ Additionally, swelling may be asymmetric with bilateral skull fractures, making it impossible to determine by imaging alone whether there has been one or multiple impact injuries.^9^ However, bilateral soft tissue swelling overlying two fracture sites typically confirms a double-impact mechanism.

An example of double-impact injury resulting in bilateral skull fractures is a fall down multiple stairs. Pennock and others showed in a retrospective analysis of stair falls in children, the high likelihood of fracture, especially when falling from a caregiver’s arms.^15^ Though long bone fractures were most common in this study, where the average age was 14.5 months, an infant rolling down multiple stairs can sustain multiple skull fractures as the head impacts multiple stairs. 

**Illustrative Case 1:**

Child Abuse Pediatrics was consulted by the Pediatric Intensive Care Unit for a 3-month-old term female who presented to the Emergency Department after reportedly falling down stairs. The mother provided a consistent history that she and the maternal aunt carried the infant unstrapped in the stroller, up the stairs at the train station, when she slipped out of the carriage head first, impacting cement stairs and rolling down 3 steps. The mother denied any loss of consciousness or emesis, and sought medical care immediately. In the Emergency Department, she was alert, well-nourished and well ap-pearing, with weight at the 75th percentile for age. Vital signs were stable with a heart rate of 138, respiratory rate of 38, and SpO2 100% on room air. Her physical exam was remarkable for biparietal soft tissue swelling with a small abrasion overlying the right scalp swelling. There was no bruising or other skin findings, no intraoral trauma, and the remaining exam was unremarkable. On psychosocial assessment, there was no past child protection involvement, no endorsement of violence in the home, and no prior injuries. Well child care was current and vaccines were up-to-date. 

**Figure 1:**
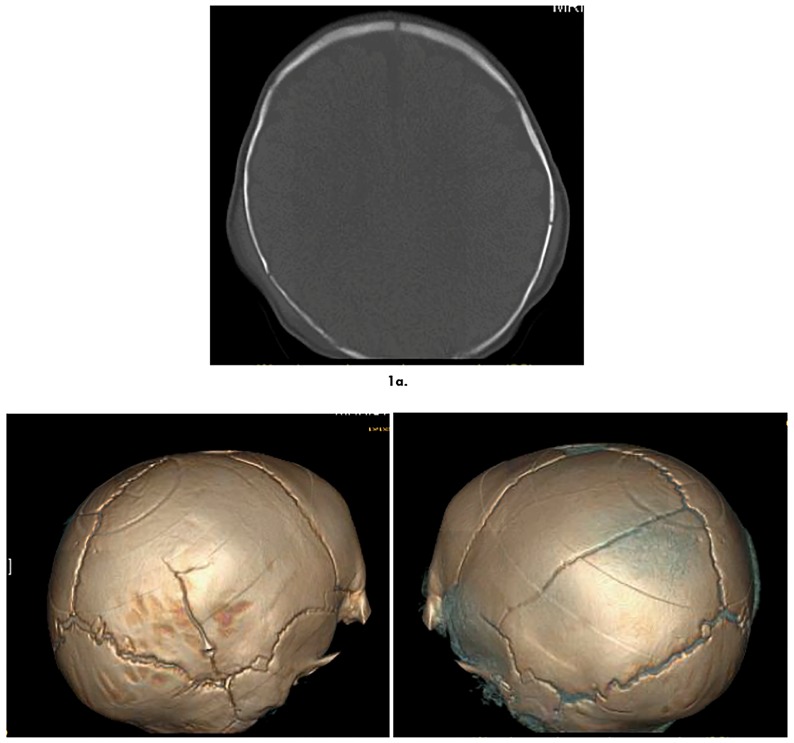
1a. Head CT coronal view demonstrating bilateral skull fracture with overlying soft tissue edema. 1b. 3D reconstruction images show asymmetric biparietal skull fractures.

Head CT with three-dimensional reconstruction imag-es is shown in Figure 1. Bilateral parietal fractures are present, each with overlying soft tissue edema. There was no intracranial hemorrhage, no mass effect, and no loss of grey-white mat-ter differentiation.

The consulting child abuse pediatrician concluded this infant experienced bilateral parietal skull fractures from an accidental fall down stairs involving multiple impact sites. Table 1 outlines recommendations for the evaluation of an infant with bilateral skull fractures, including noteworthy considerations. 

**Table 1:** Recommended evaluation for an infant with bilateral skull fractures.

Assessment	Noteworthy Features
Detailed history	Biomechanics of injury, including fall height, trajectory, and impact surface, if reported
Complete physical exam	Scalp or skin findings, oral exam, abdominal exam, extremity exam, assessing for bruising, abrasions, swelling, pain, intraoral injury, or other signs of trauma
Psychosocial Assessment	Past child protection involvement, domestic violence, prior injuries, poor well child care
Non-contrast cranial CT	Include 3-D reconstructive images
Consider complete skeletal survey	If atypical mechanism or question of abuse
Consider laboratory bloodwork	If question of abuse or medically indicated, include CBC, platelet count, coagulation studies, serum chemistries, liver and pancreatic function tests

**Compression mechanism**

Another mechanism that can result in bilateral skull fractures in an infant is compression of the head between two surfaces. This usually results in mirror-image fractures, and can be related to abuse or severe accidental injury such as a motor vehicle accident or crush injury. Hiss and Kahana performed a postmortem analysis of pediatric cases where bilateral temporoparietal fractures were caused by compression of the head between two surfaces.^16^ In one case, a 2-day-old newborn was accidentally crushed between a caregiver’s chest and door frame when he tripped with the infant in his arms, resulting in bilateral temporoparietal fractures as well as intracranial hemorrhage. In a second case, autopsy confirmed bilateral temporoparietal skull fractures, in addition to sagittal sinus laceration and intracranial hemorrhage in an 18 month old, caused by compression of the child’s head in a car doorframe. An abusive mechanism was determined in another case by the father’s confession he stomped on the head of an 8-month-old infant following a bout of persistent crying, resulting in bilateral linear skull fractures.

Another case example in the literature of bilateral skull fractures in an infant was caused by a dog bite, involving a compressive mechanism.^17^

**Single impact trauma**

Single impact trauma can also result in bilateral skull fractures that are continuous across the midline when there is impact to the cranial vertex, resulting in symmetric biparietal fractures that approximate at the sagittal suture.^9^ Additionally, single impact due to a short fall onto hard surface can occasionally result in bilateral linear skull fractures.^18^ Arnholz and others^19^ present a case whereby a witnessed accidental fall of a 6-week-old infant involving single impact to the midline occipital region from a height of 2-3 feet onto concrete, resulted in biparietal linear skull fractures. In another case report by Morse and others, a 9-month-old female sitting in a bounce seat on a countertop, pushed herself backwards, with a single impact fall 36 inches onto hardwood floor, resulting in biparietal linear fractures from a single impact event.^11^ An additional post mortem study by Weber supports that biparietal skull fractures can occur from a single impact event, due to the pliable nature of the infant skull. In this study, skull fracture patterns were assessed after infant cadavers, with median age of 3 months, were dropped from 3 feet onto different surfaces. In one case, bilateral parietal skull fractures resulted that did not approximate at the sagittal suture, in support of the theory that flexure of the pliable infant skull can lead to a second fracture away from a single impact site.^9,20^ Although these cases provide some evidence that bilat-eral skull fractures can result from single impact trauma, bilateral skull fractures without an acceptable history always warrant further evaluation for possible abuse.^9,10,11,19^

**Illustrative Case 2:**

A 10-month-old female presented to the Emergency Department with both parents reporting a fall from the mother’s arms 4 days prior. Past medical history was notable for twin gestation born at 31 weeks with pregnancy complicated by breech presentation and intrauterine growth retardation. The infant was receiving early intervention services but thriving and reaching developmental milestones consistent with her chronological age. 

On admission, the mother reported the father, a medical resident working night shifts, was sleeping in the bedroom with the infant in her crib, when she awoke crying. The mother states she picked up the infant, grabbed a diaper, and exited the room with intention to change her diaper in the living room. While holding the crying child on her right hip, with her right arm around the infant’s torso, she reports using her left arm to close the door behind her, when the infant extended her legs and arms, forcibly propelling herself off the mother’s side body. She fell backwards in an arch from the mother’s hip height (approximately 3-4 feet) onto a hardwood floor. The infant cried immediately with no loss of consciousness or emesis, and consoled with a normal feed shortly after. The next day, the mother reported first noticing swelling of the scalp while washing the baby’s hair. Ice was applied, and as she was otherwise acting well. The mother reported the swelling persisted over the next 2-3 days despite ice application, so they took the baby to the pediatrician’s office, who referred the baby to the Emergency Department.

**Figure 2:**
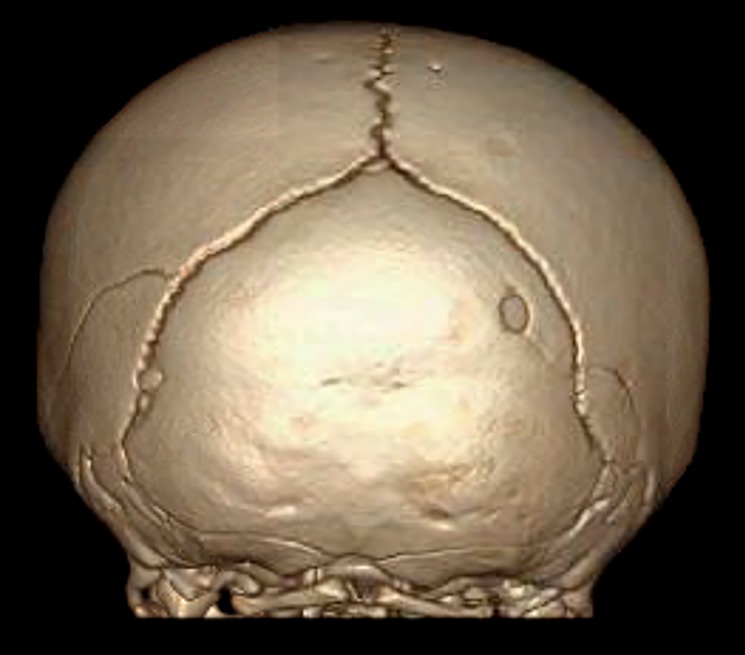
Computed tomography 3-D reconstruction showing bilateral parietal skull fractures including additional lucency on the right that crosses the lambdoid suture and communicates with a small occipital bone defect.

In the Emergency Department, the infant was alert, vigorous, and well appearing. Her weight was at the 7th percentile, temperature was 36 degrees Celsius, pulse 134, respiratory rate 18, BP 82/46 mmHg, and SpO2 100%. Exam was reported by the Emergency Physician as unremarkable but for left parietal-occipital scalp swelling measuring 8 x 8 cm. Initial skull films showed a fracture of the left parietal bone with large overlying soft tissue mass. Non-contrast head CT was obtained which showed left parietal fracture with overlying subgaleal hematoma, and additional linear fracture in the right parietal bone crossing the lambdoid suture. There was no intracranial hemorrhage or mass effect, and grey white matter differentiation appeared normal. Head CT three-dimensional skull reconstruction is shown in Figure 2.

**Figure 3:**
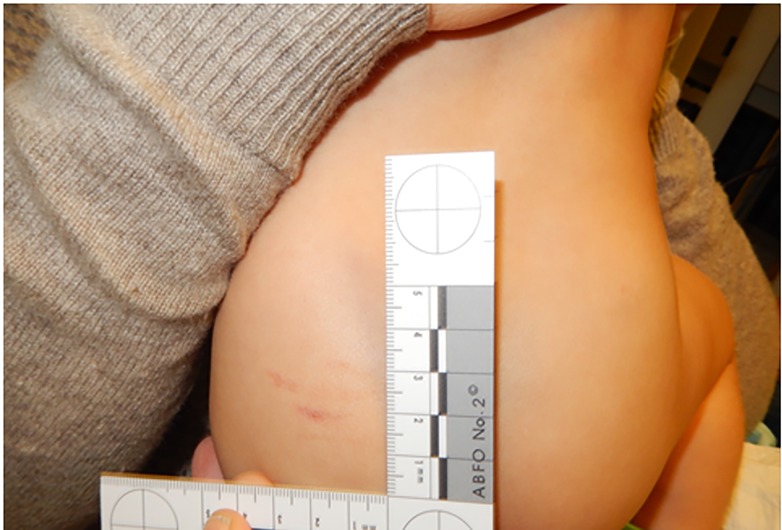
Photodocumentation of skin finding: parallel linear superficial abrasions where the mother’s hand was holding the infant, with nails scratching her as she fell, supports the accidental fall mechanism provided by the parents.

The infant was admitted for neurosurgical observation and further consultation by the child abuse pediatrician. The history remained consistent of a propelled arch occipital fall onto hard surface from mother’s right hip. Detailed skin examination was noteworthy for three linear scratch marks on the infant’s left lateral lower back, coinciding where the mother’s nails scratched the infant when he fell as described (Figure 3). There was no bruising or intraoral trauma. On psychosocial assessment, there was no past child protection involvement, no endorsement of violence in the home, and no prior injuries for this infant or the twin sibling. The infant was exclusively cared for by the mother. Well child care was current and vaccines were up-to-date.

Comprehensive skeletal survey was obtained which showed no additional fractures. The infant remained stable throughout the hospitalization, with no complications. It was concluded that the bilateral skull fractures in this infant resulted from an accidental occipital fall, involving a single impact event.

**Anatomic variants**

Whenever bilateral skull fractures are radiographically depicted without a plausible history to explain their presence, anatomic variants must be considered. These variants include a multitude of accessory sutures that may be difficult to differentiate from fracture. Plain films alone pose limitations whereby normal sutures may superimpose and mimic a fracture. Hence, if there is concern for skull fracture and inflicted head injury in children less than three years of age, it is necessary to obtain multidetector CT with 3-dimensional reconstruction of the skull for accurate diagnosis.^21,22^

The parietal and occipital bones are common sites for accessory sutures because they have multiple ossification centers. The parietal bone ossifies from two centers, while the occipital bone ossifies from 6 centers. Due to incomplete union of two ossification centers, there may be an accessory intraparietal or subsagittal suture that is usually bilateral. Both parietal and occipital accessory suture s may be unilateral but are often bilateral and fairly symmetric. ^22^ Accessory sutures have a zigzag pattern with interdigitations and sclerotic borders, whereby simple skull fractures are sharp lucencies without sclerotic edges. Distinguishing accessory sutures from true fracture may remain difficult, and may lead to false allegations of abuse. ^23,24,25,26^ In one case report of a 1-year-old male brought in for parietal soft tissue swelling without any witnessed history of trauma, bilateral symmetrical markings on plain radiographs were thought initially to be fractures. A thorough investigation for non-accidental injury revealed no additional findings, and review of the radiographs led to an accurate diagnosis of congenital subsagittal sutures with a presumed minor unwitnessed fall causing transient scalp swelling. ^24^ In another case report, a parietal skull fracture was determined to be an anomalous suture only by histologic section on autopsy, significantly altering a child protective and criminal investigation.^26^ In cases of bilateral skull fractures that may be congenital suture mimics, follow-up imaging may be necessary to determine if healing has occurred 3 months later. Because accessory sutures are commonly bilateral, they are always on the differential for any infant with bilateral skull fractures suspicious for abuse.

## Conclusion

Bilateral skull fractures may result from accidental or abusive mechanisms, involving double-impact, compression of the head between two surfaces, or single impact onto the calvarial vertex or occiput. Although bilateral skull fractures in an infant are suspicious for abuse, a thorough evaluation, sometimes involving consultation with a child abuse specialist, may assist with consideration of plausible mechanisms of injury in conjunction with the history provided by the caregiver, to determine if suspicious for abuse. Accessory sutures that mimic fracture, especially if the fracture lines appear symmetric, must also be considered. Three-dimensional reconstruction of CT bone images aids the diagnosis and should be routinely performed on all infants with findings concerning for bilateral skull fractures. Future studies involving retrospective or prospective data may further inform our understanding of mechanisms of injury that can result in bilateral skull fractures in infancy.
